# The effect of Working Alliance on drug attitude in patients with Major Depressive Disorder

**DOI:** 10.1192/j.eurpsy.2022.1423

**Published:** 2022-09-01

**Authors:** G. Santarelli, G. Sanfilippo, F. Benvenuti, L. Santoro, A. Nistri, M. Innocenti, A. Ballerini, V. Ricca

**Affiliations:** 1 University of Florence, Human Health Sciences, Firenze, Italy; 2 University of Florence, Human Health Sciences, firenze, Italy

**Keywords:** Working Alliance, Drug attitude, beliefs about medicines, major depressive disorder

## Abstract

**Introduction:**

Working Alliance is defined as the emotional bond and the agreement on therapeutic goals and tasks between patients and therapists. Despite the wide use of the construct of working alliance in research on psychotherapy, few studies have investigated the role of working alliance in influencing adherence to pharmacotherapy, and drug attitude. A deeper knowledge of the interplay between working alliance and drug attitude could help to challenge low adherence to psychopharmacological treatments in Major Depressive Disorder.

**Objectives:**

This study aimed to investigate the relationship between working alliance and drug attitude in patients with Major Depressive Disorder.

**Methods:**

27 patients admitted in the Psychiatric Unit of Careggi with diagnosis of Major Depressive Disorders were enrolled. Working Alliance Inventory - patient version (WAI-P), Drug Attitude Inventory (DAI) and Beliefs about Medicines (BMQ) were administered. Pearson’s correlation was used to assess relationships between variables.

**Results:**

A significant positive correlation was detected between BMQ total scores, DAI total scores and WAI-P task, bond, and goal subscales.

**Table:** 

Correlations between WAI-P subscales and BMQ and DAI total scores				
	DAI total scores		BMQ total scores	
	r	p	r	p
**WAI-P task**	0.551	**0.003**	0.613	**0.001**
**WAI-P bond**	0.430	**0.001**	0.560	**0.004**
**WAI-P goal**	0.621	**0.001**	0.603	**0.002**

**Conclusions:**

Such preliminary data suggest a relationship between Working Alliance and drug attitude. This could contribute to provide tools to challenge low adherence to psychopharmacological treatments in patients with Major Depressive Disorder.

**Disclosure:**

No significant relationships.

